# AI-assisted assessment and treatment of aphasia: a review

**DOI:** 10.3389/fpubh.2024.1401240

**Published:** 2024-08-29

**Authors:** Xiaoyun Zhong

**Affiliations:** School of Humanities and Foreign Languages, Qingdao University of Technology, Qingdao, China

**Keywords:** AI, aphasia, aphasia assessment, aphasia treatment, people with aphasia

## Abstract

Aphasia is a language disorder caused by brain injury that often results in difficulties with speech production and comprehension, significantly impacting the affected individuals’ lives. Recently, artificial intelligence (AI) has been advancing in medical research. Utilizing machine learning and related technologies, AI develops sophisticated algorithms and predictive models, and can employ tools such as speech recognition and natural language processing to autonomously identify and analyze language deficits in individuals with aphasia. These advancements provide new insights and methods for assessing and treating aphasia. This article explores current AI-supported assessment and treatment approaches for aphasia and highlights key application areas. It aims to uncover how AI can enhance the process of assessment, tailor therapeutic interventions, and track the progress and outcomes of rehabilitation efforts. The article also addresses the current limitations of AI’s application in aphasia and discusses prospects for future research.

## 1 Introduction

Aphasia is a language disorder that arises from brain damage. It can be caused by neurodegenerative diseases, but mainly occurring after strokes, where it affects approximately 21–42% of survivors (1). Aphasia typically results from damage to one or more language areas in the brain, predominantly in the left hemisphere where critical language functions reside. Hence, people with aphasia (PWA) may experience a wide range of impairments across different language domains, including language production, comprehension, and reading and writing. The impact on each of these areas can vary depending on the location and extent of the brain damage, leading to diverse manifestations of the disorder. For example, Wernicke’s Aphasia is a type of fluent aphasia where PWA can speak fluently but their speech often lacks meaningful content, and this aphasia is typically associated with damage to the posterior part of the left temporal lobe. Anomic Aphasia, on the other hand, primarily involves difficulty in naming people or things, which is usually linked to damage in the left temporoparietal area.

Although aphasia is a communication disorder that significantly impairs an individual’s ability to convey and understand language and not a mental disorder itself, its impact can lead to challenges in emotional and social domains, resulting in psychological and social difficulties. Research indicates that 93% of PWA experience substantial psychological distress following a stroke, a rate significantly higher than that observed in stroke survivors without aphasia (2). Additionally, approximately half of PWA suffer from anxiety (3), and depression is prevalently noted as a common psychological issue within this group (4). These emotional challenges have been identified as detrimental to the health-related quality of life for PWA (5). A survey involving 75 diseases and 66,000 patients indicated that aphasia has the most significant negative impact on quality of life, far exceeding that of Alzheimer’s disease and quadriplegia (6).

Consequently, precise assessment and appropriate therapeutic interventions are crucial for effective management of aphasia. Typically, the care of aphasia includes several main stages: initial assessment, targeted treatment, continuous monitoring, and adaptive therapies based on PWA’s progress. The initial assessment comprehensively evaluates PWA’s language abilities, determining aphasia classification and severity, with an aim to provide appropriate treatment (7). Targeted treatment might involve speech therapy and cognitive exercises aimed at restoring language functions. Continuous monitoring ensures that changes in PWA’s condition are promptly addressed, allowing for adjustments in therapy (8). After that, adaptive therapies may be implemented to better help PWA recover.

It is evident that assessment and treatment stand as the pivotal components. An accurate assessment is vital as it allows healthcare professionals to pinpoint the specific type and severity of the condition, which is essential for determining the most suitable treatment to foster recovery. Most aphasia assessments are made using language tests, which can be inadequate (9) and time-consuming. There is a pressing need for innovative techniques to improve this process. In rehabilitation, similarly, due to constraints in medical and economic resources, it is often necessary to integrate intensive language training with additional methods to maximize its effectiveness (10). Techniques such as transcranial direct current stimulation (tDCS) and virtual reality (VR), for example, are among these adjunct approaches that can significantly enhance therapeutic outcomes (11).

In recent years, artificial intelligence (AI) has been gradually introduced into this field by optimizing both the assessment and treatment for aphasia. Recent reviews have explored the application of AI in the context of aphasia, each with a distinct focus. For example, Azevedo et al. (12) and Adikari et al. (13) conducted scoping reviews on the use of AI in aphasia diagnosis and rehabilitation, emphasizing the current research landscape in these areas. In contrast, Privitera et al. (14) examined the ethical and practical considerations surrounding the application of AI in aphasia. Despite the valuable insights provided by previous reviews, there remains a significant need for narrative reviews in the application of AI in aphasia, which facilitate a deeper exploration of specific topics. This may be helpful in a complex field like aphasia, where the interplay of technological advancements and human experiences must be understood. Therefore, this paper aims to provide a more in-depth analysis of the application of AI in aphasia, particularly focusing on how different AI technologies are applied in assessment and treatment.

This paper begins with a brief overview of AI and its key applications in managing aphasia in Section 2. Section 3 reviews previous research related to how AI has been used in aphasia assessment. A review on how AI has been applied in the treatment of aphasia is presented in Section 4. In Section 5, there is discussion on the present challenges facing AI in this area and directions suggested for future research. Finally, the conclusion is presented in Section 6.

## 2 AI technology and its application in aphasia

AI refers to the simulation of human intelligence by machines, involving the development of algorithms, which are sets of rules designed to perform specific tasks or solve problems. AI technology enables computers to perform tasks typically requiring human intelligence, such as learning, reasoning, problem-solving, perception, and language understanding (15). The evolution of AI started with Symbolic AI, which utilized rule-based systems. This was followed by the era of Machine Learning (ML). ML is a branch of artificial intelligence that shifted focus to data-driven algorithms (16), where systems learn from data to make predictions or decisions without being explicitly programmed. The development of deep learning (DL) further refined this approach. DL is a subset of ML that uses neural networks with many layers to analyze and learn from large amounts of data. DL’s advantage lies in using multi-layered neural networks for complex tasks like image and speech recognition (17).

In the field of language, significant progress has been made in natural language processing (NLP) and understanding. NLP is a cornerstone of AI that allows computers to perform automated analysis of text by applying linguistic principles. This technology is crucial for extracting essential information about language disorders from texts (18), laying the groundwork for intelligent question-answering systems and related functionalities. Recently, techniques such as Chatbots and speech recognition have revolutionized how people interact with machines using language. AI-powered language models like GPT-4 have demonstrated remarkable capabilities in generating human-like text and answering complex questions.

AI has a wide range of applications, and its role in healthcare, particularly in managing neurological diseases, is significant (19), offering groundbreaking solutions that range from predictive analytics to personalized medical care. Specifically, in the field of neurology, AI is making significant advances in the assessment and treatment of neurological diseases such as Alzheimer’s, Parkinson’s, multiple sclerosis, and epilepsy (20). AI’s primary role in managing neurological disorders stems from its capacity to analyze extensive data sets, provide early predictions, and facilitate precise diagnoses and interventions (20, 21). For example, utilizing AI algorithms enables the early prediction of seizures in epilepsy (22). By analyzing large amounts of medical data, AI algorithms can identify patterns that might not be noticeable to humans.

AI is making remarkable progress in aphasia research. The powerful capabilities of ML, DL, NLP, and advanced algorithms are also laying the groundwork for various technological innovations (13). For example, DL has significantly advanced speech recognition technology, improving its ability to identify complex patterns in speech signals. These developments are highlighting and facilitating the integration of this technology into both the study and treatment of aphasia, demonstrating AI’s potential to make a meaningful impact in this field (23, 24). By combining ML, DL, speech recognition, and NLP, AI is starting to show its potential in diagnosing and rehabilitating aphasia.

## 3 AI-based aphasia assessment

The clinical assessment of aphasia is typically conducted through aphasia scales, with commonly used scales including the western aphasia battery (WAB) (25) and the Boston diagnostic aphasia examination (BDAE) (26). These scales are primarily designed to assess key language skills including auditory comprehension, spontaneous speech, naming, and writing. Their purpose is to pinpoint the specific nature of the language disorder and gage its severity. Yet, these conventional tools for diagnosing aphasia are not without their drawbacks; they often involve lengthy procedures and can yield inconsistent results when comparing outcomes from different scales (27). Against this backdrop, the introduction of AI technology offers new possibilities for the assessment of aphasia, especially in determining aphasia severity and classification. AI-assisted aphasia assessment is mainly achieved by speech signal processing, speech recognition and transcription, and image analysis. The primary content and conclusions of such studies can be seen in Table 1.

**Table 1:** Summary of studies on aphasia assessment.

Authors, year	Target of assessment	Methods/Models	Language	Sample size	Aphasia severity	Accuracy	Correlation with human assessment	Accuracy/correlations influenced by severity
Mahmoud et al. 2020 (30)	Speech lucidity features	High-resolution time-frequency images with a convolutional neural network	Mandarin	12 PWA	NP		Articulation 0.71; fluency 0.60; tone scores 0.58	Yes
Khan et al. 2017 (29)	Aphasia type	Digital signal processing	NP	15 PWA	NP	100%		NP
Metu et al. 2023 (28)	Fluency	Convolutional and recurrent neural networks	NP	22 PWA	from mild to severe	63.6% (non-fluent) and 81.8% (fluent)		NP
Qin et al. 2020 (31)	Aphasia classification	2-layer gated recurrent unit (GRU) and CNN	Cantonese	91 PWA	from mild to severe	62–92% (CNN); 53–83% (GRU)		NP
Lee et al. 2016 (32)	Assessment of voice and speech disorders	Gaussian mixture model-hidden Markov model (GMM-HMM); deep neural Network-hidden Markov model (DNN-HMM)	Cantonese	17 PWA	Aphasia Quotient (AQ): 73:2–99:0	41.8% (GMM-HMM); 42.2% (DNN-HMM)		Yes
Qin et al. 2018 (16)	Aphasia severity	Bag-of-word (CBOW) model; DNN-HMM	Cantonese	104 PWA	AQ: 11–99		0.84	NP
Le et al. 2018 (33)	Speech recognition; aphasia severity	Bidirectional long-short term memory recurrent neural network (BLSTM-RNN)	English	401 PWA	From mild to very severe	62.63% for speech recognition accuracy; 0.8 for correlation in predicting AQ		Yes
Nivedha et al. 2023 (23)	Aphasia severity	Hybrid attention inception ResNetV2-based chaotic slime mold (HAIR-CSM)	Cantonese	91 PWA	From low AQ to high AQ	98.10%		NP
Perez et al. 2020 (24)	Speech recognition	Mixture of Experts (MoE), DNN acoustic model	NP	300 PWA	From mild to severe	63%		Yes
Qin et al. 2019 (35)	Speech recognition; aphasia severity	Time-delay neural network combined with BLSTM (TDNNBLSTM)	Cantonese	92 PWA	AQ: 11–99		0.83	Yes
Le and Prost 2016 (36)	Speech recognition	GMM-HMM; DNN-HMM	English	401 PWA	From mild to very severe	60.30%		Yes
Themistocleous et al. 2021 (37)	Aphasia classification	NLP	English	52 PWA	NP	64–77%		NP
Day et al. 2021 (38)	Aphasia severity	NLP	English	238 PWA	From mild to very severe	73%		Yes
Fraser et al. 2014 (39)	Aphasia assessment and classification	NLP	English	24 PWA	NP	70–90% (aphasia recognition); 60% (classfication)		NP
Pustina et al. 2017 (41)	Aphasia severity	Stacked multimodal prediction (STAMP)	English	53 PWA	AQ: 30–98		0.79–0.88	NP
Moral-Rubio et al. 2021 (43)	Aphasia assessment and classification	ML models for EEG	NP	40 PWA	NP	75% (aphasia recognition); 58% (classfication)		NP
Krishna et al. 2021 (44)	Aphasia assessment	DL models for EEG	English	9 PWA	AQ: 48–94.8	50%		NP
Kristinsson et al. 2021 (45)	Aphasia severity	ML models for neuroimaging data	NP	116 PWA	Mean AQ: 62.9	67% (AQ); 61% (fluency); 66% (spontaneous speech); 53% (naming); 65% (repetition); 61% (auditory comprehension)		NP
Jeong et al. 2022 (46)	Aphasia severity	DL model for neuroimaging data	Korean	176 PWA	From mild to very severe		0.59–0.72	NP
Matias-Guiu et al. 2019 (47)	Aphasia classification	ML models in comparison with neuroimaging data	NP	68 PWA	NP	86–89%		NP
Landrigan et al. 2021 (48)	Aphasia classification	ML models for neuroimaging data	English	226 PWA	AQ: 25.5–97.9	75%		NP

### 3.1 Speech signal processing

Speech signal processing typically involves analyzing the acoustic features of speech sounds to identify communication-related details. In the field of aphasia, the application of speech signal processing provides an avenue for a comprehensive examination of various acoustic features of speech such as tone and frequency. By applying advanced techniques to analyze these features, AI enables a precise and quick understanding of the individual linguistic characteristics manifested in aphasic speech. For example, Metu et al. (28) employed convolutional neural networks (CNN) to parse PWA’ acoustic spectrograms, focusing on identifying characteristics like disfluency and pauses to detect non-fluent aphasia. Simultaneously, they used recurrent neural networks (RNN) to assess the semantic coherence of speech within aphasic contexts, helping to pinpoint fluent aphasia. Their algorithm demonstrated an accuracy exceeding 81%, aligning with therapists’ diagnoses of fluent and non-fluent aphasia at rates of 81.8 and 63.6%. Likewise, using methods of feature extraction and pattern matching, it was demonstrated feasible to differentiate between anomic and Wernicke’s aphasia based on a mix of acoustic characteristics such as formants, combined with language features and time consumed (29).

Initially, the focus of speech signal processing predominantly catered to PWA speaking Indo-European languages. Recently, its application has extended to encompass those people who speak other languages, such as Mandarin and Cantonese (30, 31). For example, in a study employing the Cantonese Aphasia Bank, which includes spontaneous speech recordings from post-stroke PWA, it was discovered that even with varied acoustic models, syllable error rates reached up to 58.2 and 57.8% for different models (32). This suggests that language remains a key factor in achieving high precision in this field. However, in another study Mandarin speech spectrograms were transformed into detailed time-frequency images that function as training data for machine learning models, which allowed for the assessment of aphasia severity through analysis of articulation, fluency, and tonal qualities of speech and demonstrated a high correlation (30).

### 3.2 Speech recognition and transcription

Speech recognition involves converting spoken language into analyzable textual information. Speech recognition technology stands as a cornerstone in both the research and clinical assessment of aphasia, serving as an essential instrument for spotting and automatically assessing speech difficulties in PWA (16). By analyzing key language aspects such as information density, fluency, vocabulary richness, and structural complexity, speech recognition technology allows for a detailed assessment that contributes to the estimation of the WAB scores (33).

The progress in AI boosts the accuracy of speech recognition, aided by the implementation of algorithms such as deep learning, which further refines its precision. Despite variations in the assessment metrics employed in different studies, evidence indicates that speech recognition technology can discern signs associated with aphasia effectively. For instance, symptoms indicating aphasia can be accurately identified with an impressive rate of 98.1% accuracy (23). In contrast, previously the application of speech recognition in diagnosing aphasia faced challenges with high error rates, surpassing even 70% (34). Recent developments have seen significant improvements, with phoneme recognition error rates decreasing to 37% for moderate aphasia (24) and syllable recognition error rates to 38.4% (35). Nevertheless, for more severe cases of aphasia, the error rates associated with speech recognition technology still tend to be considerably high, even exceeding 75% (36).

Additionally, AI digs deeper into the verbal output of PWA and can transcribe PWA’s speech to conduct further analysis. In this field, NLP acts as an impressive asset, autonomously classifying the components of speech in the language generated by PWA. For example, such analysis assists in distinguishing between primary progressive aphasia (PPA) subtypes through an evaluation of the usage ratio of different words (37). Another study used NLP to examine the length and lexical diversity of sentences produced by PWA (38). The researchers developed a machine learning model capable of measuring the severity of aphasia, demonstrating a high degree of precision with an average absolute error below 7% and an overall accuracy exceeding 73%, reaching up to 87.5% for mild aphasia cases. Similarly, Fraser et al. (39) extracted linguistic features like word frequency, sentence length, and noun-to-verb ratios from PWA’s speech. It turned out that their algorithm successfully categorized individuals into control group, PPA group, and semantic dementia group. The algorithm attained accuracy levels ranging from 70 to 90% for distinguishing between patients and control subjects, and over 60% for identifying the patient subgroups.

### 3.3 Image analysis

When diagnosing aphasia, AI technologies are frequently being integrated with neuroimaging and Electroencephalogram (EEG) data. This powerful combination lends significant assistance to therapists (40–42). In particular, the role of image analysis proves pivotal in accurately diagnosing aphasia, as it multidimensionally visualizes brain activities, facilitating a comprehensive understanding of this condition. Research by Moral-Rubio et al. (43) has shown that machine learning algorithms, when applied to resting-state EEG data, can distinguish between individuals with PPA and control groups with a 75% accuracy rate.

Crucially, the implementation of AI algorithms enables the integration of data obtained from image analysis with other forms of clinical data. This integration substantially enhances the accuracy and efficacy of aphasia diagnoses. For example, integrating speech and EEG signals from PWA during reading tasks has been shown to enhance algorithm performance by up to 50% (44). Kristinsson et al. (45) have developed a predictive model using machine learning that combines functional magnetic resonance imaging, brain lesion volume, and other data types, achieving an accuracy rate of over 60% in evaluating the severity of aphasia in PWA. Moreover, deep learning can merge with linguistic data linked to aphasia to gage aphasia severity (31). Jeong et al. (46) delved into the capabilities of Deep Feed-Forward Networks (DFFNs), which are foundational to deep learning and commonly applied in image-centric tasks. Their research aimed to predict the intensity of aphasia in individuals who were in the early stages of acute stroke. To this end, they examined brain lesions that showed up in magnetic resonance diffusion-weighted imaging and analyzed additional clinical data. A correlation of 0.72 between DFFN-generated predictions and actual WAB scores was obtained from their findings, which shows the immense potential of AI in the exploration of clinical image.

Furthermore, by involving the technique of image analysis, AI research has unlocked subtler types of aphasia. For example, Matias-Guiu et al. (47) explored the use of machine learning algorithms in conjunction with brain imaging data to identify subtypes of PPA. Their research unveiled that the non-fluent and logopenic strains of aphasia could be each further classified into subcategories, yielding a total of five distinct PPA subtypes. This insight challenges the traditional classification of PPA, which acknowledges only three subtypes, and enriches the understanding of this disorder. A further study combined statistical methods, machine learning, and neuroimaging tools to sort PWA using 20 different data types, including WAB scores and the frequency of semantic errors (48). The results highlighted that, beyond those with milder aphasia, PWA could be distinctly grouped based on whether they had difficulties with phonetic or semantic processing. This AI-driven categorization showed a 75% correspondence with brain neuroimaging findings, surpassing the traditional classification into fluent and non-fluent types, which matches brain imaging only about 60% of the time. This insight hints at the potential shortcomings of conventional aphasia classification methods.

## 4 AI-assisted aphasia rehabilitation

By pushing past the temporal and spatial barriers in traditional therapy and promoting autonomous training, AI plays an increasing role in rehabilitation studies. For example, Azevedo et al. (12) have noted that AI is increasingly being utilized as a central component in augmentative and alternative communication devices. However, a closer examination of AI’s role in rehabilitation reveals that it not only personalizes treatment plans for PWA, but also monitors their progress and delivers adaptive therapies. In addition, the predictive ability of AI makes the treatment results foreseeable.

### 4.1 Aphasia treatment

#### 4.1.1 Evaluation and feedback

Providing prompt evaluation and effective feedback on the results of training sessions is a significant advantage of AI-assisted rehabilitation therapies. Continually training machine learning models with the speech of PWA can improve the machine’s evaluation of aphasic speech (49), thereby better assisting PWA in rehabilitation. Furthermore, AI technology can be integrated with specific equipment and technologies to assist communication for PWA, such as incorporating speech recognition technology into devices like iPads (50, 51), thus providing automatic feedback and enabling PWA to undertake self-directed rehabilitation training.

The effectiveness of AI-assisted treatment, including its evaluation system and feedback mechanisms, also relies heavily on the processes of speech signal processing and speech analysis. AI-based technologies are capable of evaluating and giving feedback on the quality of PWA’s speech output. Le et al. (52) developed a model with machine learning trained with speech data from PWA. This model is capable of evaluating PWA’ speech based on fluidity, clarity, effort, and prosody, delivering results that are on par with manual evaluations. Treatment tools as such enable the provision of immediate evaluation and feedback on rehabilitation training without necessitating the presence of a therapist.

Besides the quality of aphasic speech, AI-assisted treatment tools are able to judge the content of aphasic speech. Barbera et al. (53) employed deep learning technologies to create a naming judgment system. Its algorithm enables a quick comparison between the speech of PWA and their healthy counterparts, swiftly determining the accuracy of PWA’ naming efforts and providing feedback with an accuracy rate surpassing 84%. Research has also shown that for PWA who have writing disorders the technology of speech recognition can also be of assistance. For example, the program Dragon NaturallySpeaking is capable of transforming the speech of PWA into text. Following a training period, the recognition accuracy of this program surpassed 84% (54), thereby facilitating PWA in written communication. The use of this program can also stimulate the recovery of some language abilities, such as producing speech with fewer errors, higher coherence, and the BDAE scale also shows that PWA’s oral repetition ability has improved (55).

#### 4.1.2 Virtual interaction

**Figure 1:**
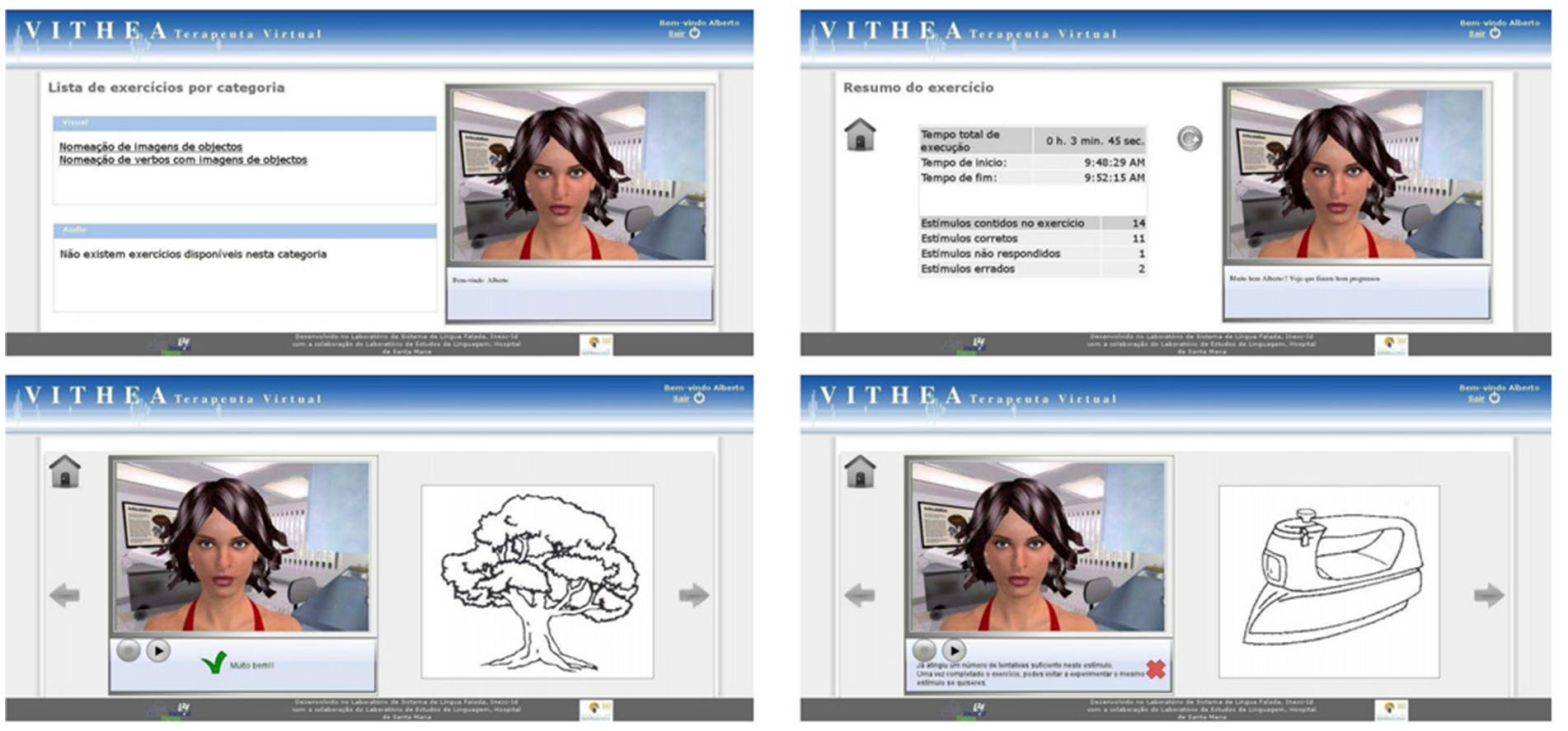
Operation interface of VITHEA (56).

Advancements in AI have led to the creation of virtual therapists, a groundbreaking tool in aphasia treatment. They exist at the intersection of technologies like speech recognition, natural language processing, and machine learning. This enables them to engage in live interaction with PWA. For example, Abad et al. (56) developed a virtual therapist named virtual therapist for aphasia treatment (VITHEA). Figure 1 provides a view of VITHEA, with its user-friendly interface designed for PWA and the combination of an avatar alongside the training material (56). The avatar simulates interaction and guides users through exercises. The left or right section lists exercise categories, for example, visual images with options for naming objects and audio sounds if necessary. This online system simulates a therapist providing language exercises for PWA and gives feedback based on the PWA’s responses. Human therapists can also use this system to adjust exercise methods or track PWA’s rehabilitation progress.

Another similar application is the web-based oral reading for language in aphasia (ORLA) program (57), a particular therapy that has been adapted for computer and online usage. ORLA guides PWA through repeated reading of sentences or paragraphs by virtual therapists, either independently or in remote collaboration with human therapists, who can monitor the treatment process. Compared to traditional treatment, ORLA has been shown to have better long-term effects (58), and the therapeutic efficacy of web-based ORLA is comparable to that of ORLA treatments conducted by human therapists (59). In addition to training focused on specific language units, virtual therapists can also help simulate real-world communication scenarios for treatment. For instance, Kalinyak-Fliszar et al. (60) used script training in virtual interaction, allowing PWA to engage in conversations with the virtual therapist on specific topics, and PWA turned out to be willing to communicate with the virtual therapist and experienced positive treatment outcomes.

**Figure 2:**
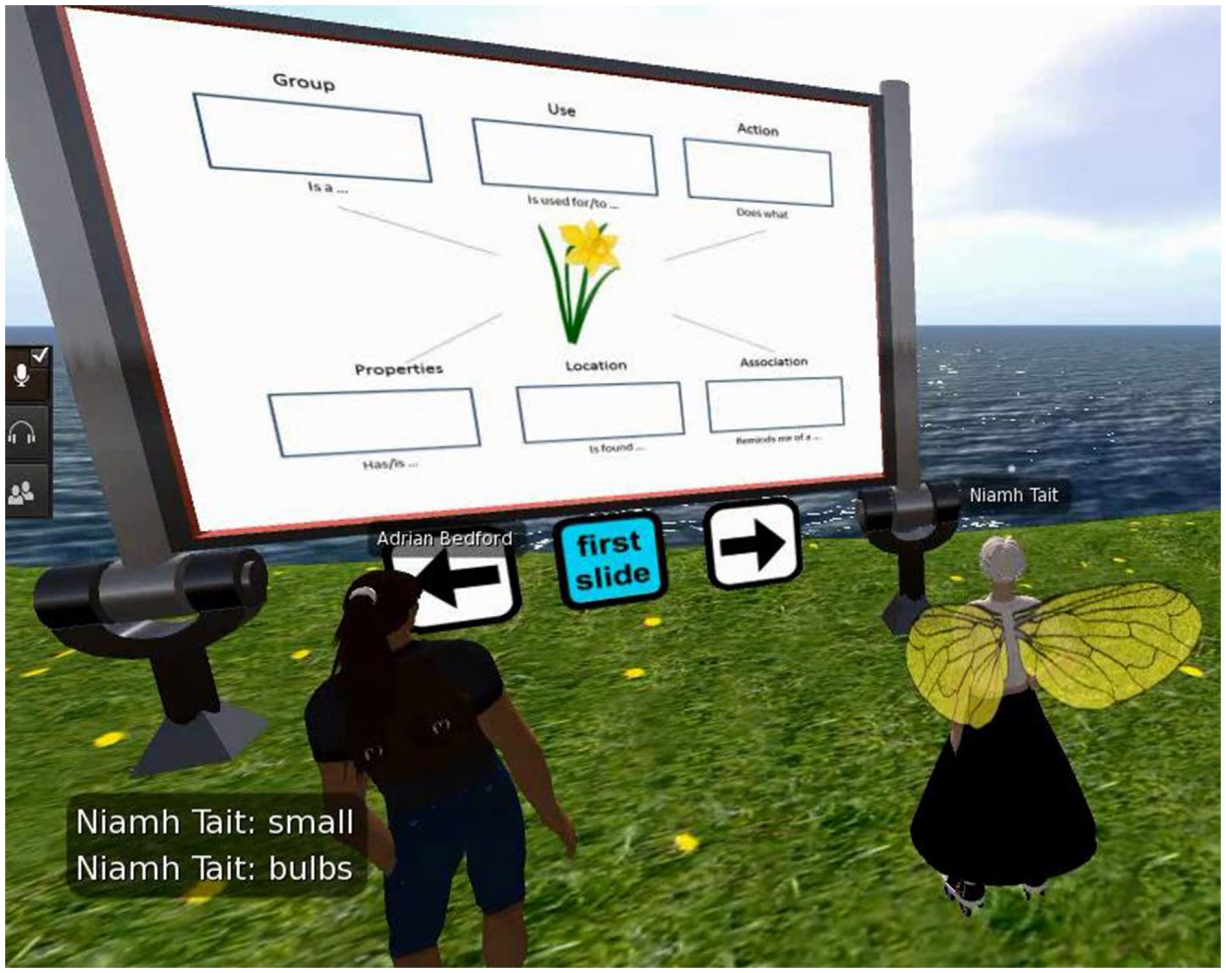
The EVA park interface (virtual avatars for both the individual with aphasia and the therapist) (64).

Virtual reality (VR) is also playing a transformative role in aphasia treatment, integrating a wide array of AI technologies, which include deep learning algorithms for user motion, and environmental and behavioral simulations, alongside natural language processing for sophisticated dialog. The majority of studies applying VR in aphasia treatment have demonstrated positive results (61). One particularly prominent VR platform is the EVA Park (62) tailored for PWA. It provides a multi-user virtual space, enabling PWA to interact with therapists, their fellows, and tech support in real-time. EVA Park creates a range of lifelike scenarios, for example in town squares and cafes, and offers a dynamic backdrop for training tasks. EVA Park guides PWA toward achieving treatment effects with activities modeled after EVA Park’s realistic environments, such as practicing to make requests within a health center or hair salon (63). Figure 2 offers a snapshot of an interactive session between a user with aphasia and a virtual therapist (64). The user and the virtual therapist are conversing amidst natural scenery, simulating real-world communication scenarios to aid in language skill practice, with language materials displayed in a slide format. In this case, the slide focuses on naming a certain object, describing its features, and practicing the use of these words in various sentences. Studies utilizing EVA Park have applied language tasks including naming (64) and storytelling (65), all signifying enhanced linguistic capabilities in participants. A significant advantage of this approach over conventional language aphasia therapies is the increased level of PWA’s engagement. Research indicates that EVA Park significantly boosts PWA’s desire for interaction and fosters positive emotional states (66), with these beneficial impacts enduring over time (67).

### 4.2 Outcome prediction

AI technology is also emerging as a potent tool in forecasting the outcomes of aphasia recovery. Aphasia treatment may yield limited success or in certain instances fail entirely, and the underlying causes of these variations still remain largely unexplored (68–70). Furthermore, given the intricate nature of aphasia, where each person responds differently to therapy, this presents a considerable hurdle. Within the realm of AI, the application of specialized computational models, when integrated with detailed PWA data, offers a promising solution to predict recovery outcomes and even unearth the factors influencing them.

The prediction about aphasia recovery is usually accomplished by integrating large datasets and multiple data sources. For example, research by Saur et al. (71) revealed that it is possible to forecast PWA’s language proficiency 6 months post-stroke by merging brain imaging data with PWA’s linguistic characteristics and age, where data-driven models were used for analysis and categorization. In a similar vein, Gu et al. (72) applied algorithms to analyze over 130 attributes related to PWA’s brain structures and behavioral data during treatment, and their model predicted aphasia recovery outcomes with a 74% accuracy rate. This model pinpointed essential distinctions among PWA with different levels of rehabilitation success. Billot et al. (73) employed a similar approach to gather data on PWA, finding that predictive models based on a wide array of data, incorporating brain imaging and WAB scores, can precisely estimate the response rate to treatments in people with chronic-phase aphasia, achieving a 90% accuracy. The data identified as most vital for forecasting the results of rehabilitation were resting-state brain connectivity, brain tissue integrity, and the severity of aphasia.

Applying AI technologies also makes it possible to simulate recovery process and thus predict recovery outcomes, especially for bilingual PWA (74). Bilingual PWA often exhibit varying degrees of impairment across their two languages (75), and the potential for overlapping linguistic generalization in treatment remains a topic in debate, which adds complexity when formulating treatment strategies. Grasemann et al. (76) developed computational models trained on extensive English and Spanish vocabulary to replicate the process of acquiring semantic and phonological components of language in bilingual individuals. They also conducted treatments based on lexical semantic analysis with bilingual PWA and simulated the treatment process using computational models. The findings suggested that computational models driven by AI are not only capable of simulating and predicting the impact of treatment on PWA but also identify the generalization effects of the treated language on the untreated language. Therefore, the predictive capabilities of AI can offer reference in selecting the target language of treatment for bilingual PWA (77), potentially enhancing treatment efficiency and reducing costs.

## 5 Discussion

AI is increasingly acknowledged for its transformative role in aphasia assessment and rehabilitation. With its ability to autonomously process and analyze extensive language data, AI not only improves assessment accuracy and customizes rehabilitation programs to individual needs but also helps conserve medical resources and streamline the care process. The innovations driven by AI technology are paving the way for enhanced diagnostic criteria and more effective rehabilitation methods, marking a significant shift in care approaches. However, despite these advancements, the integration of AI into aphasia care is still in its early stages (28). Addressing this will require focused efforts across multiple critical areas.

Despite the promising advancements in AI for aphasia assessment, there remains a significant challenge in matching the assessment accuracy of experienced human clinicians. This gap highlights the urgent need for further innovation and refinement in AI algorithms. Achieving unmatched assessment precision requires enhanced sensitivity and specificity of AI algorithms. To enhance AI algorithms, it is crucial to integrate more advanced machine learning techniques, which can better model the complexity of human language. Furthermore, collaboration with linguistic experts and continuous training with diverse, real-world datasets will help improve the algorithms’ ability to detect and understand the subtle speech patterns specific to aphasia. Additionally, incorporating a broader spectrum of clinical data, such as genetic information (78), can enrich AI’s learning environment and foster a more sophisticated understanding of aphasia.

The integration of AI in aphasia rehabilitation holds promise, but as pointed out by Privitera et al. (14), currently, AI falls short in addressing the emotional needs of PWA. This gap highlights the necessity for AI systems to incorporate empathetic design and emotional intelligence features, ensuring that the technology not only aids in communication recovery but also supports the psychological wellbeing of PWA. To address this issue, integrating AI’s analytical strengths with the attentive and compassionate care provided by healthcare professionals may offer a viable solution. Establishing a collaborative environment where AI tools and healthcare professionals work together creates an atmosphere that is both data-informed and sensitive to the emotional and social needs of each individual with aphasia. This holistic approach can enrich the assessment and rehabilitation practice, ensuring that the clinical environment is more adaptive, comprehensive, and ultimately more effective.

Applying AI in aphasia requires a collaborative, multidisciplinary approach. The intricate nature of aphasia necessitates research and treatment strategies that are as diverse as the condition itself. To tackle aphasia’s complexities, it is essential to integrate expertise from the specialized yet interrelated fields of medicine, linguistics, neuroscience, and AI technology. Promoting interdisciplinary collaboration allows for the unique strengths and insights of each field to be merged. Such a united approach is vital in creating assessing and therapeutic strategies that are not only effective but holistic, addressing the diverse and intricate needs of PWA.

Additionally, the future of treating aphasia may experience further transformation thanks to the integration of generative language models like Chat-GPT (13). These advanced models are paving the way for innovative, personalized language rehabilitation activities. They may enhance traditional therapy by providing interactive, real-time conversations. As these models get better at understanding and replicating human speech, they could provide precise and relevant interactions. This not only helps in developing communication skills but also offers a support system that complements the efforts of human therapists, ensuring continuous assistance beyond clinical settings. This blend of cutting-edge technology with speech pathology is set to make aphasia treatment more effective.

## 6 Conclusion

In sum, AI holds immense promise for revolutionizing aphasia assessment and treatment. Machine learning algorithms, for instance, are capable of examining extremely large datasets of speech patterns to detect subtle differences and variations, thereby facilitating the early and precise assessment of aphasia. Additionally, AI-driven applications can be tailored to deliver personalized treatment, dynamically adjusting to the progress and challenges faced by PWA. AI’s capacity to continuously learn and adapt promises significant advances in effectiveness of aphasia treatment. By harnessing real-time data and evolving with patient responses, it is possible that in the future AI can perpetually refine therapy techniques to better meet the needs of PWA.

However, there are significant limitations to consider. AI systems often lack the precision needed for fine-grained understanding of language, which is essential in therapeutic contexts. Moreover, these systems may fall short in addressing the emotional and social needs of patients, which are vital components of effective communication therapy. Despite these challenges, the potential of AI to revolutionize aphasia care is immense, offering a future where technology and human expertise converge to provide solutions that are more effective, accessible, and tailored to individual needs.
